# Effectiveness of Nutrition and Exercise Interventions Among Community-Dwelling Older Vietnamese Adults with Sarcopenia: A Cluster-Randomized Controlled Trial

**DOI:** 10.3390/nu18183080

**Published:** 2026-09-20

**Authors:** Tan Duy Doan, Huyen Thi Thanh Le, Tuan Nhat Pham, An Thanh Truong, Huong Thi Le

**Affiliations:** 1 Faculty of Public Health, University of Medicine and Pharmacy at Ho Chi Minh City, Ho Chi Minh City 72723, Vietnam; phamnhattuan@ump.edu.vn (T.N.P.); thanhan@ump.edu.vn (A.T.T.); 2 School of Preventive Medicine and Public Health, Hanoi Medical University, Hanoi 116177, Vietnam; lethihuong@hmu.edu.vn; 3 HCMC Hospital for Rehabilitation—Professional Diseases, Ho Chi Minh City 73023, Vietnam; lethithanhhuyen032002@gmail.com; 4 Department of Clinical Nutrition, Vietnam National Cancer Hospital, Hanoi 11018, Vietnam

**Keywords:** sarcopenia, older adults, nutritional support, exercise intervention, muscle strength, physical performance

## Abstract

Background/Objectives: Exercise and nutrition are central to sarcopenia management, but Vietnamese evidence is limited. We compared exercise intervention (Ex) alone and Ex plus nutritional support against health education. Methods: In this study, a three-arm, parallel-group cluster-randomized controlled trial, three communities in Can Gio District were randomly selected and assigned 1:1:1 (one per arm) to health-education control, Ex, or combined intervention. Before allocation, 30 adults aged 60–70 years with AWGS 2019-defined sarcopenia were randomly selected from screening lists within each community (*n* = 90). Both active arms underwent 24 weeks of aerobic and resistance exercise, with weekly supervised sessions during weeks 1–12 and less-supervised home exercise thereafter. The combined arm also received weekly individualized nutrition counseling and a daily multinutrient oral supplement (270 kcal; 11 g protein) during weeks 1–12. Outcomes at baseline, 12 weeks, and 24 weeks were analyzed using sex-adjusted participant-level linear mixed-effects models. Results: At 24 weeks, adjusted differences in change (95% CIs) for Ex versus control and combined versus control were, respectively, 0.12 (0.02–0.22) and 0.01 (−0.09–0.11) kg/m^2^ for the skeletal muscle index; 2.49 (1.57–3.42) and 2.23 (1.30–3.15) kg for handgrip strength; and 0.206 (0.132–0.279) and 0.053 (−0.017–0.123) m/s for gait speed. Conclusions: Both active communities showed better handgrip strength than control; only the Ex community showed a gait-speed advantage, while muscle-mass changes were modest. With one cluster per arm, treatment and community were confounded; definitive treatment effects and the independent contribution of nutrition cannot be determined. Trial registration: ClinicalTrials.gov NCT07761091 (retrospectively registered on 12 August 2026).

## 1. Introduction

Sarcopenia is characterized by the progressive loss of skeletal muscle mass (SMM) and strength with aging and is associated with impaired mobility, fractures, disability, hospitalization, and mortality [1,2,3,4,5,6,7]. The Asian Working Group for Sarcopenia (AWGS) 2019 provided diagnostic criteria widely used in Asian populations [2]. The AWGS 2025 consensus further broadened the focus from diagnosis to lifelong muscle health, emphasizing early identification and integrated strategies to preserve muscle function [1]. Moreover, AWGS has emphasized early identification and intervention in community settings, where functional decline may still be modifiable [1,2].

Vietnam is experiencing rapid population aging, and sarcopenia has become an important community health concern. Recent Vietnamese studies have reported a substantial prevalence of sarcopenia among community-dwelling older adults, although estimates differ according to age structure and measurement methods [8,9]. Low body weight, frailty, low physical activity, and socioeconomic factors have been associated with sarcopenia in these settings [8,9].

Nutrition is integral to that muscle-health framework. Older adults need sufficient energy to avoid catabolic loss and sufficient high-quality protein to support muscle protein synthesis, while malnutrition, poor appetite, weight loss, and low nutrient intake can accelerate functional decline [10,11,12,13,14]. Asian and international guidance, therefore, places nutritional assessment and individualized nutrition care alongside exercise in sarcopenia management rather than treating nutrition as an optional adjunct [10,11,12,13].

Exercise and nutrition act through complementary pathways. Resistance and multicomponent exercise provide the mechanical and neuromuscular stimulus needed to improve strength and physical performance [15,16,17,18,19,20], whereas adequate energy and amino-acid availability support the adaptive response to training [11,20,21,22,23]. Protein-based supplements, vitamin D, beta-Hydroxy-beta-methylbutyrate (HMB)-containing formulations, and individualized dietary strategies have shown benefit in selected older populations, especially when nutritional vulnerability is present [24,25,26,27,28,29,30,31,32,33,34].

The magnitude of benefit from combined interventions is heterogeneous. Recent meta-analyses generally support exercise-plus-nutrition strategies for sarcopenia, but the response differs according to baseline diet and nutritional status, age, BMI, intervention dose, supplement composition, and duration [16,17,18,35,36]. These findings support evaluating nutritional care as part of a multimodal strategy while recognizing that its incremental effect may vary across populations and intervention designs.

Despite the growing recognition of sarcopenia, evidence from community settings in low- and middle-income countries—such as Vietnam—remains limited. In particular, there is a scarcity of data on individuals with AWGS-specific sarcopenia identified through population screening rather than in specialist clinical settings. To address these gaps, we evaluated a pragmatic, community-based, three-arm intervention among older adults in Can Gio District, Ho Chi Minh City. The study compared health education alone (control), health education plus structured exercise intervention (Ex), and the same Ex program combined with individualized nutrition counseling and nutrient-rich oral nutritional supplementation (ONS). Notably, the intervention also incorporated a subsequent phase to observe the active cessation of nutritional support.

This exploratory three-arm cluster-randomized trial aimed to characterize changes from baseline to weeks 12 and 24 in SMI (primary outcome), HGS, and 6 m gait speed (secondary outcomes) among community-dwelling older adults with AWGS 2019 sarcopenia [2]. We compared the observed trajectories of a health-education community, a community receiving the same education plus structured exercise, and a community receiving exercise plus individualized nutrition counseling and ONS during the first 12 weeks. Baseline energy and protein intake are presented only to describe the nutritional context of the study population. Because only one community was assigned to each arm, between-group comparisons are interpreted as exploratory.

## 2. Materials and Methods

### 2.1. Study Design and Setting

This study was a three-arm, cluster-randomized community trial conducted among community-dwelling older adults with sarcopenia in Can Gio District, Ho Chi Minh City, Vietnam. Can Gio is a geographically dispersed coastal suburban district with rural and semi-urban communities. The community (township/commune) was the unit of randomization and the individual older adult was the unit of observation. One community was assigned to each study arm: (1) control, receiving health education; (2) Ex, receiving health education plus an Ex program; or (3) combined, receiving health education, the Ex program, individualized nutrition counseling, and ONS.

Outcomes were assessed at baseline (T0), after 12 weeks (T1), and after 24 weeks (T2). T1 represented the end of the active nutrition-support period. From weeks 13 to 24, the Ex program continued with less direct supervision, whereas scheduled nutrition counseling and ONS were discontinued in the combined group. Reporting was prepared with reference to CONSORT 2025, the CONSORT extension for cluster-randomized trials, and the TIDieR framework [37,38,39].

### 2.2. Participants

#### 2.2.1. Preceding Community Screening and Sampling Frame

The intervention cohort was drawn from a preceding community screening study. That screening used a two-stage cluster sampling strategy. In the first stage, the sampling frame comprised one township and six communes in Can Gio District, representing 7206 older adults listed by local older adult associations. Three clusters were selected by simple random sampling using Stata: Can Thanh Township, Binh Khanh Commune, and Long Hoa Commune. Together, these communities included 4283 listed older adults.

**Figure 1:**
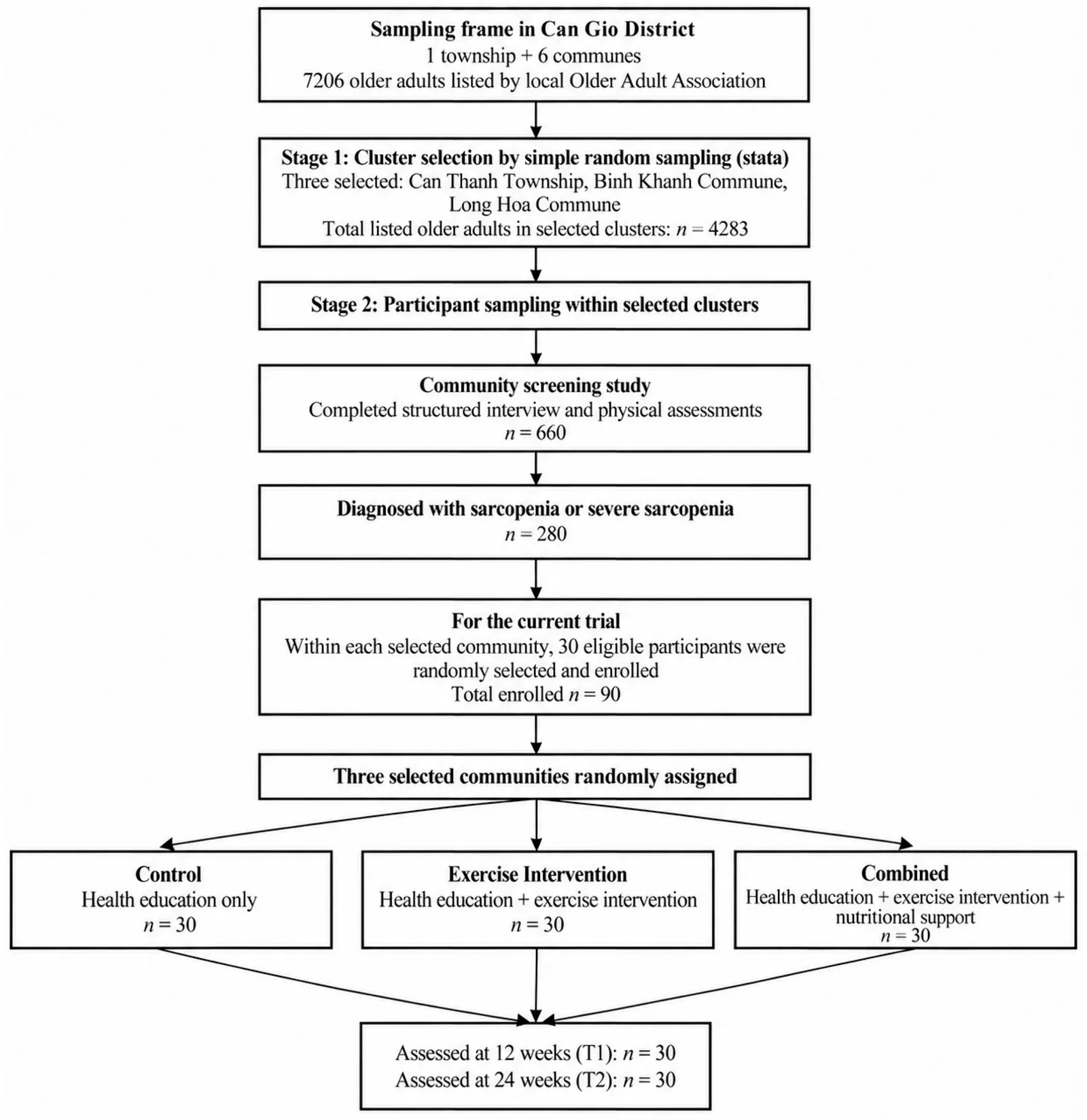
Sampling, community allocation, and participant flow from the preceding screening study to the 24-week intervention follow-up.

In the second stage of the screening study, the number of residents sampled from each selected community was allocated in proportion to the size of the local older-adult population. Individuals were then selected by simple random sampling from resident lists in collaboration with local health workers and older adult associations. Selected residents were approached in the community and invited to complete structured interviews and standardized physical assessments. The screening study yielded an analytic cohort of 660 older adults, of whom 280 met AWGS 2019 criteria for confirmed sarcopenia or severe sarcopenia (99 with sarcopenia and 181 with severe sarcopenia) [2] (Figure 1).

#### 2.2.2. Cluster Allocation and Trial Participant Recruitment

After the three communities had been selected, they were randomly allocated in a 1:1:1 ratio to the study conditions using Stata by an independent team member who was not involved in participant recruitment, intervention delivery, or outcome assessment. Binh Khanh Commune was allocated to the control condition, Can Thanh Township to Ex, and Long Hoa Commune to the combined intervention. The community allocation sequence was held independently and disclosed only after community selection had been completed (Figure 1).

For the intervention trial, the screening database was restricted to participants aged 60–70 years with confirmed sarcopenia or severe sarcopenia who met the trial eligibility criteria, including being able to walk independently, being able to participate safely in the Ex program, and providing written informed consent. Thirty participants were then selected by simple random sampling from each community-specific eligible list and invited to join the intervention for a total of 90 participants.

Exclusion criteria were participation in another trial or nutrition/exercise program likely to influence the outcomes; active cancer or progressive malignancy; chronic kidney disease requiring dialysis; acute or unstable disease requiring priority treatment; poorly controlled cardiopulmonary disease that made exercise unsafe; musculoskeletal disease, recent injury, or surgery that substantially limited mobility; prolonged systemic corticosteroid use, weight-loss medication, or unstable thyroid hormone therapy; and contraindication, allergy, or intolerance to the ONS used in the study.

### 2.3. Sample-Size Calculation

The original calculation targeted the planned combined-versus-control comparison for a muscle-mass outcome. Post-intervention skeletal muscle mass reported by Wang et al. (13.88 ± 2.39 versus 16.42 ± 2.98 kg) corresponded to an estimated standardized difference of d ≈ 0.94 [32]. With two-sided α = 0.05 and 90% power, *n* = 2(Z_1−α/2_ + Z_1−β_)^2^/d^2^ yielded approximately 24 participants per group; allowing 20% attrition increased the target to 30 per group (90 total). This participant-level calculation did not account for cluster design, three-arm comparisons, or a prespecified clinically important SMI difference. Consequently, the achieved sample should be considered adequate only for the large effect assumed in the original calculation, not for modest treatment differences or community-level causal inference.

### 2.4. Blinding and Bias-Reduction Procedures

Participants and intervention providers could not be blinded because the interventions involved exercise, counseling, and supplementation. Outcome assessors were separated from the intervention team and were not informed of community allocation. Participants were reminded not to disclose their intervention during assessments. Study-arm labels were coded during initial data cleaning and analysis. The same measurement procedures and equipment were used across study arms and assessment waves.

### 2.5. Interventions

All three groups received health education. The Ex group additionally received the structured Ex program, while the combined group received the same Ex program together with individualized nutrition counseling and ONS.

At week 12, both exercise communities had completed the more intensively supervised phase, and the combined community had also completed scheduled nutrition counseling and daily oral supplementation. From weeks 13 to 24, exercise continued with direct supervision once a month, whereas the nutrition program was discontinued. The week-24 combined-community trajectory, therefore, represents a sequential intervention rather than 24 weeks of concurrent exercise and nutrition. Attenuation after week 12 cannot be attributed specifically to withdrawal of nutritional support because supervision intensity, adherence, and other unmeasured exposures also changed.

#### 2.5.1. Health Education

A group-based health-education session was delivered at the beginning of the intervention by nutritionists from the University of Medicine and Pharmacy at Ho Chi Minh City. The session addressed nutrition and exercise for the prevention and management of sarcopenia and was supported by a handbook and videos. Health education was provided to all three study groups.

#### 2.5.2. Exercise Intervention

The exercise program was developed and supervised by a physical education lecturer holding a master’s degree at Ho Chi Minh City University of Education. It comprised music-based aerobic exercise for 30 min and resistance training targeting the major muscle groups for 30 min per session. Two 60 min exercise sessions were prescribed each week for 24 weeks. The exercises are demonstrated in two Vietnamese-language instructional videos provided to participants (available at https://youtu.be/73vmL7u8D1Y, accessed on 17 August 2024 for resistance exercises, and at https://youtu.be/aUsks685jN4, accessed on 17 August 2024 for aerobic exercises). During weeks 1–12, participants attended one directly supervised session each week and completed the second prescribed session at home using the exercise handbook and videos. During weeks 13–24, one session per month was directly supervised, while all other prescribed sessions were performed at home. The supervising lecturers reviewed exercise technique and individualized progression according to each participant’s functional capacity and tolerance.

#### 2.5.3. Nutritional Support

Nutritional support was provided only to the combined group during weeks 1–12 and was designed as individualized nutrition care rather than supplementation alone. Nutritionists delivered counseling once weekly through a combination of face-to-face and remote contacts. Counseling was tailored to nutritional status, habitual dietary intake, and comorbidities and focused on adequate energy intake, total protein, high-quality protein sources, distribution of protein across the day, dietary balance, and management of gastrointestinal symptoms. Participants also received one serving of ONS daily for 12 weeks. Each serving provided approximately 270 kcal, 11 g protein, 0.6 g HMB, 0.3 g omega-3, 2.0 g omega-6, 5.25 g omega-9 fatty acids, and 300 IU vitamin D. Scheduled counseling and ONS were stopped after week 12, whereas Ex continued through week 24. The detailed intervention components by study arm and phase is shown in Table 1.

### 2.6. Outcomes and Outcome Measurements

Study outcomes were assessed at baseline (T0), 12 weeks (T1), and 24 weeks (T2). Change in SMI from baseline to week 24 was selected as the primary outcome because low appendicular muscle mass was an eligibility-defining component of AWGS 2019 [2] sarcopenia and was a biologically relevant target of resistance exercise and nutritional support. HGS and gait speed were evaluated as secondary outcomes representing muscle function and physical performance. Their potentially greater responsiveness does not justify changing the primary outcome after results were known.

#### 2.6.1. Body Composition and Skeletal Muscle Index

Appendicular SMM was measured using the same multifrequency bioelectrical impedance analysis (BIA) (InBody 770, InBody Co., Ltd., Seoul, Republic of Korea) at all visits by trained dietitians following a common operating procedure. The subjects were instructed to avoid exercise 6–12 h before the test, avoid eating 3–4 h before the test, avoid consuming alcohol and coffee 12 h before the test, and avoid using body lotion on the palms or soles of the feet before the test. During the test, the interviewer asked the subjects to remove all socks, tights, shoes, thick clothing (jackets), and metal items (jewelry, watches, belts) [40].

SMI was calculated as appendicular SMM divided by height squared (kg/m^2^). Low MM at enrollment was defined using the AWGS 2019 BIA cut-offs of SMI < 7.0 kg/m^2^ in men and <5.7 kg/m^2^ in women [2]. Body weight, BMI, SMM, fat-free mass (FFM), and fat mass (FM) were assessed at the same visits. Measurements were performed under standardized conditions regarding recent food and fluid intake, physical activity, bladder emptying, clothing, and time of assessment as far as feasible. Equipment functionality was checked before field sessions, and comparable procedures were used at T0, T1, and T2.

Post-intervention categorical reclassification according to AWGS 2019 [2] was not evaluated; therefore, the proportion of participants who no longer met sarcopenia criteria at 12 or 24 weeks could not be determined.

#### 2.6.2. Handgrip Strength

Muscle strength was assessed using HGS measured with a Camry Digital Handgrip Dynamometer, model EH101 (Zhongshan Camry Electronic Co. Ltd., Zhongshan, China) and expressed in kilograms. Assessors received standardized training on participant positioning, instructions, equipment operation, recording, and safety. The same study operating procedure was used at all three assessments. According to AWGS 2019, low muscle strength was defined as HGS < 28 kg in men and <18 kg in women [2]. Change in HGS was calculated as the follow-up value minus the T0 value. Therefore, a positive change indicated improvement in muscle strength.

#### 2.6.3. Walking Performance

Physical performance was assessed with a 6 m walking test. Gait speed (m/s), the registered physical-performance metric, was calculated as 6 m divided by transit time in seconds. Higher values indicate better physical performance. According to AWGS 2019, low physical performance was defined as gait speed < 1.0 m/s [2]. The same 6 m testing procedure was used at T0, T1, and T2.

### 2.7. Baseline Characteristics

Habitual physical activity outside the study intervention was not quantified using a validated physical-activity questionnaire or an objective activity monitor.

#### 2.7.1. Sociodemographic and Social Characteristics

Personal and social characteristics obtained through the interview included sex (male/female), age, educational background (under primary school, primary school, secondary school, high school or above), health insurance participation (yes or no), employment status (yes/no), low family economics (yes/no, based on participant self-report), cohabitation (yes/no), family support (yes/no), and social activities (yes/no).

#### 2.7.2. Clinical Characteristics

Multimorbidity was assessed using the Charlson Comorbidity Index, with a total score of ≥2 indicating the presence of multiple comorbid conditions [41,42,43]. Polypharmacy was defined as the concurrent use of five or more medications (≥5 medications) [44] and was recorded as a binary variable (yes/no).

#### 2.7.3. Dietary Assessment

Baseline dietary intake was assessed by trained dietitians using a food-frequency questionnaire for one month. Food photographs were used to estimate portion size. Reported foods and beverages were converted to energy and protein using the Vietnamese food-composition database. The estimates were collected before exposure to the intervention and are presented descriptively. Dietary intake was not reassessed at weeks 12 or 24; therefore, intervention-related dietary change and individual energy or protein adequacy could not be evaluated.

### 2.8. Adherence and Safety

Intervention delivery used the standardized materials and schedules described above. Quantitative variables for supervised-session attendance, home-exercise completion, ONS consumption, counseling attendance, and adverse events were not included in the locked effectiveness dataset used for this manuscript. Accordingly, adherence, dose–response, and safety are not analyzed quantitatively here, and the absence of such results should not be interpreted as evidence of complete adherence or absence of adverse events.

### 2.9. Statistical Analysis

Continuous variables were summarized as mean ± standard deviation (SD), and categorical variables as number and percentage. Baseline comparisons used appropriate tests according to variable type, with Fisher’s exact test where required.

For body-composition outcomes and HGS, longitudinal patterns were analyzed with sex-adjusted linear mixed-effects models fitted by restricted maximum likelihood. Models included fixed effects for study group, time, and the group × time interaction and a participant-level random intercept. Within-group contrasts compared T1 and T2 with T0. Exploratory pairwise contrasts estimated differences in change for Ex versus control, combined versus control, and combined versus Ex, with 95% confidence intervals (CIs). Wald tests were used for model-based inference.

Given the small and markedly unequal numbers of men across communities and the absence of replicated clusters within study arms, sex-by-group interaction effects were not estimated because such analyses would be unstable and difficult to interpret. Sex was therefore retained as an adjustment covariate.

Because baseline gait speed differed materially among communities, gait-speed change was calculated as follow-up gait speed minus baseline gait speed for T1 and T2. These change scores were analyzed using a linear mixed-effects model fitted by restricted maximum likelihood, with fixed effects for study group, follow-up time, the group × time interaction, sex, and baseline gait speed, and a participant-level random intercept. Table 4 reports adjusted mean changes from baseline with 95% CIs and within-group *p*-values (p_w_). Overall, between-group differences at each follow-up (p_b_) were assessed using omnibus Wald tests. Table 5 reports the corresponding adjusted pairwise differences in change for Ex versus control, combined versus control, and combined versus Ex. Positive estimates indicate a greater increase in gait speed in the first-listed group.

Participants were analyzed according to the intervention assigned to their community. Because only one randomized community represented each study arm, intervention condition was completely confounded with community. Community-level variance and the intracluster correlation coefficient could therefore not be estimated. Participant-level confidence intervals and *p*-values may consequently understate uncertainty attributable to cluster allocation. All between-group contrasts are therefore interpreted as exploratory descriptions of observed between-community patterns, with emphasis on the direction, magnitude, confidence intervals, and consistency of estimates across time rather than definitive causal treatment effects. Analyses were performed in Stata 17.0 (StataCorp. 2021. Stata: Release 17. Statistical Software. StataCorp LLC.: College Station, TX, USA).

### 2.10. Ethics Approval and Consent to Participate

The study was approved by the Institutional Review Board of Hanoi Medical University (approval No. 1078/GCN-HMUIRB, dated 4 January 2024). All participants were informed about the study objectives and procedures, and written informed consent was obtained before data collection. Personal information was kept confidential and used only for research purposes.

The trial was retrospectively registered on ClinicalTrials.gov as NCT07761091, entitled “Effectiveness of Nutrition and Exercise Interventions in Older Adults with Sarcopenia in Can Gio District, Ho Chi Minh City”. Because registration was retrospective, the registration record cannot be used to establish prospective specification of the exact primary endpoint or statistical analysis model.

## 3. Results

Among the 660 older adults in the preceding community screening cohort, 280 (42.4%) met AWGS 2019 [2] criteria for confirmed or severe sarcopenia: 99 of 660 (15.0%) had sarcopenia and 181 of 660 (27.4%) had severe sarcopenia. The intervention trial subsequently enrolled 90 eligible adults aged 60–70 years, with 30 participants selected from each of the three study communities.

### 3.1. Study Participant Characteristics

The mean age was 65.7 ± 3.3 years in the control group, 65.0 ± 3.1 years in the Ex group, and 65.7 ± 3.4 in the combined group. The control community had a higher proportion of men (46.7%) than the Ex (16.7%) and combined (20.0%) communities. Baseline energy intake ranged from 1338.5 to 1479.6 kcal/day, while mean protein intake was similar across groups (61.5–62.7 g/day), indicating broadly comparable starting protein intake at the group level. Baseline characteristics are shown in Table 2.

### 3.2. Baseline Muscle-Related Outcomes

Baseline body weight ranged from 51.8 to 52.9 kg and BMI from 21.6 to 22.7 kg/m^2^. SMI was 5.7 ± 0.6 kg/m^2^ in the control group, 5.5 ± 0.5 kg/m^2^ in Ex, and 5.4 ± 0.6 kg/m^2^ in the combined group. HGS was 17.2 ± 7.9, 17.2 ± 6.8, and 19.2 ± 5.3 kg, respectively. Baseline gait speed was 0.79 ± 0.19 m/s in the control community, 0.95 ± 0.19 m/s in Ex, and 0.87 ± 0.18 m/s in the combined community (Table 3).

### 3.3. Comparison of Body Composition, Muscle Strength, and Gait Speed Before and After Intervention

Body-composition changes were modest over 24 weeks (Table 4). At T1, body weight increased by 0.6 kg (95% CI: 0.1 to 1.1) in Ex and by 0.5 kg (95% CI: 0.1 to 1.0) in the combined group, while the overall group-by-time test was not significant. FM, FFM, and SMM showed no consistent separation among the three communities.

**Figure 2:**
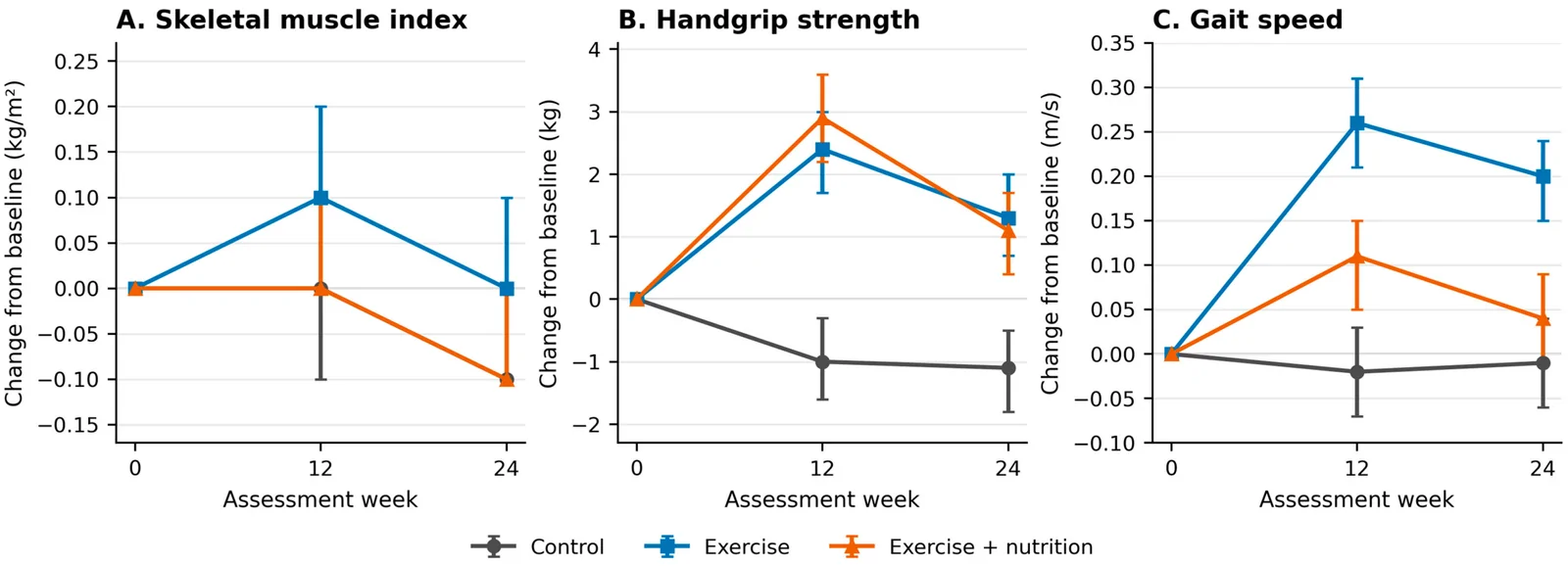
Model-estimated changes from baseline in (**A**) skeletal muscle index, (**B**) handgrip strength, and (**C**) 6 m gait speed at 12 and 24 weeks. Points show adjusted mean changes and error bars show 95% confidence intervals. All models were adjusted for sex. Gait-speed estimates were additionally adjusted for baseline gait speed. Baseline is anchored at zero because the plotted values are changes from baseline. With one community per arm, intervals do not incorporate community-level variation and the trajectories should be interpreted as exploratory community patterns.

SMI changed little in absolute terms. The overall group-by-time test was not significant at T1 (p_b1_ = 0.173) and reached statistical significance at T2 (p_b2_ = 0.041). In exploratory pairwise analysis at T2, the difference in SMI change was 0.12 kg/m^2^ (95% CI: 0.02 to 0.22, *p* = 0.021) for Ex versus control, 0.01 kg/m^2^ (−0.09 to 0.11, *p* = 0.792) for combined versus control, and −0.10 kg/m^2^ (−0.20 to −0.00, *p* = 0.041) for combined versus Ex (Table 5) (Figure 2). These participant-level contrasts are descriptive of the observed community patterns and should not be interpreted as definitive treatment-effect estimates. These participant-level contrasts describe the observed differences among the three intervention communities and should not be interpreted as unbiased treatment-effect estimates.

HGS showed a clear functional response in both intervention communities. At T1, HGS changed by −1.0 kg (95% CI −1.6 to −0.3) in control, +2.4 kg (95% CI: 1.7 to 3.0) in Ex, and +2.9 kg (95% CI: 2.2 to 3.6) in the combined group. At T2, the corresponding changes were −1.1 kg (−1.8 to −0.5), +1.3 kg (95% CI: 0.7 to 2.0), and +1.1 kg (95% CI: 0.4 to 1.7). Exploratory pairwise differences in change favored both Ex and combined intervention versus control at both follow-ups. HGS did not clearly separate the two active interventions (combined versus Ex: 0.52 kg, 95% CI: −0.41 to 1.44 at T1; −0.27 kg, 95% CI: −1.19 to 0.66 at T2) (Figure 2).

Gait speed showed a strong early response in both active communities (Table 4). At T1, the baseline- and sex-adjusted mean change was −0.02 m/s (95% CI: −0.07 to 0.03) in control, 0.26 m/s (95% CI: 0.21 to 0.31) in Ex, and 0.11 m/s (95% CI: 0.06 to 0.15) in the combined group. At T2, adjusted changes were −0.01 m/s (95% CI: −0.06 to 0.04), 0.20 m/s (95% CI: 0.15 to 0.24), and 0.04 m/s (95% CI: −0.01 to 0.09). In pairwise analyses at T2, the adjusted difference in change was 0.21 m/s (95% CI: 0.13 to 0.28) for Ex versus control, 0.05 m/s (95% CI: −0.02 to 0.12) for combined versus control, and −0.15 m/s (95% CI: −0.22 to −0.08) for combined versus Ex (Table 5) (Figure 2).

## 4. Discussion

This community-based study compared structured Ex alone and the same Ex program combined with individualized nutritional support against health education in older adults with sarcopenia. Functional outcomes showed the clearest changes. HGS improved in both active communities relative to control, whereas the gait-speed response differed over time: both active communities improved at 12 weeks, but the improvement was more sustained in the Ex community at 24 weeks. Body-composition changes were small. At 24 weeks, the combined-versus-control estimate for SMI was close to zero, while the Ex community showed a modestly higher SMI change than control. These findings should be interpreted as exploratory community patterns rather than definitive component effects because each intervention was represented by a single community.

The separation between functional gains and small changes in MM is consistent with the biology of early training adaptation. Strength and mobility can improve through neural adaptation, motor-unit recruitment, coordination, and changes in muscle quality before measurable hypertrophy is apparent [45,46,47,48,49]. A 2026 systematic review of 72 exercise studies in older adults with sarcopenia likewise found more consistent benefits for strength and physical performance than for MM [36]. Function and mass should therefore be interpreted as complementary, rather than interchangeable, outcomes.

The functional response observed in both active communities is compatible with current evidence supporting resistance and multicomponent exercise as a central component of sarcopenia care [15,16,17,18,19,20,35,36]. In our program, direct supervision was more frequent during the first 12 weeks and then shifted toward a maintenance phase. HGS and walking improvements were largest at T1 and were smaller at T2. Reduced supervision, changes in adherence, or insufficient progression of the exercise stimulus may have contributed, but these mechanisms cannot be separated with the available data. The combined community received individualized counseling and daily oral supplementation only during weeks 1–12. Although its handgrip-strength trajectory was favorable relative to control, it did not show a consistent additional advantage over exercise alone. This comparison cannot isolate nutritional efficacy because there was no nutrition-only arm, one community represented each condition, dietary change and adherence were unavailable, and nutrition was discontinued while exercise continued. The appropriate interpretation is therefore uncertainty about the incremental contribution of this specific 12-week nutrition strategy, not evidence that nutrition is ineffective in sarcopenia care.

Recruitment and diagnosis were based on AWGS 2019, the applicable Asian consensus when the study was designed. AWGS 2025 reframes sarcopenia within a broader life-course muscle-health model and explicitly supports multimodal management that integrates resistance exercise and nutritional support [1,2]. This updated framework strengthens the clinical rationale for assessing nutritional status and tailoring nutrition alongside Ex, while future trials should prospectively examine whether response differs according to nutritional vulnerability and updated AWGS-defined profiles.

The study has several practical strengths. Participants came from a population-based community screening program, were randomly selected from community-specific eligible lists, and received interventions that could be delivered through local health systems. The combined intervention used individualized counseling in addition to ONS, which more closely reflects nutrition practice than supplementation alone. Outcomes were measured repeatedly over 24 weeks by assessors who were separated from intervention delivery.

Several limitations should be considered. First, only three communities were randomized, with one community assigned to each study arm. Consequently, intervention condition was completely confounded with community-level effects, and participant-level confidence intervals and *p*-values may underestimate uncertainty attributable to cluster allocation. Second, the original sample-size calculation was performed at the participant level and did not account for clustering or multiple three-arm comparisons; therefore, the study was not demonstrably powered to detect modest clinically meaningful between-group differences or to support community-level causal inference. Third, baseline characteristics were imbalanced across communities, particularly sex distribution and gait speed. Although sex was included as a covariate and gait-speed models were additionally adjusted for baseline gait speed, these adjustments cannot fully eliminate residual confounding. Baseline habitual physical activity was also not quantitatively characterized. Fourth, quantitative adherence and adverse-event data were unavailable, precluding evaluation of intervention exposure, dose–response relationships, and safety. Dietary intake was assessed only at baseline; therefore, longitudinal changes in energy and protein intake and their relationships with muscle outcomes could not be evaluated. Fifth, body composition was assessed using BIA, and the small observed changes in SMI should be interpreted cautiously because BIA measurements may be influenced by hydration and other premeasurement conditions. Sixth, the trial was retrospectively registered on ClinicalTrials.gov (NCT07761091); therefore, the registration record cannot verify prospective specification of the primary outcome or statistical analysis plan. Finally, the study included ambulatory adults aged 60–70 years from a single district, which may limit generalizability.

Future studies should incorporate adequate treatment-level replication by including multiple communities per study arm or, where feasible, using individual randomization, together with prospective registration of the study protocol and statistical analysis plan. To better determine the independent and incremental contribution of nutritional support, future trials should consider a nutrition-only arm or a factorial design and provide nutritional care for a duration appropriate to the expected biological response. Nutritional assessment should prospectively characterize baseline nutritional status and risk, energy adequacy, protein intake relative to body weight, and meal-level protein distribution, while also monitoring longitudinal dietary intake, supplement and counseling adherence, exercise adherence, habitual physical activity, and adverse events. Future trials should also prospectively evaluate categorical changes in AWGS-defined sarcopenia status alongside continuous changes in muscle mass, strength, and physical performance. Such studies would allow more reliable estimation of intervention effects and help identify individuals most likely to benefit from individualized nutritional support as part of multimodal sarcopenia care.

## 5. Conclusions

In this exploratory three-community trial, both active intervention communities showed better HGS than the control community, while gait speed improved most clearly and was sustained through 24 weeks in the Ex community. The combined community also showed an early gait-speed improvement at 12 weeks, but the adjusted change was smaller at 24 weeks. Body-composition changes were modest. These results do not isolate the independent contribution of nutritional support and should not be interpreted as evidence against nutrition, because nutritional care was embedded within the combined program and was provided for only 12 weeks, and the study had one community per arm. Larger prospectively registered trials with multiple clusters per arm, complete nutrition and adherence measures, and sustained nutritional follow-up are needed to quantify the contribution of individualized nutritional support within multimodal sarcopenia care.

## Figures and Tables

**Table 1:** Intervention components by study arm and phase.

Intervention Component	Control Group	Ex Group	Combined Group
Health education	One group session at start	One group session at start	One group session at start
Aerobic exercise	-	30 min/session, 2 sessions/week, weeks 1–24	Same as Ex
Resistance exercise	-	30 min/session, 2 sessions/week, weeks 1–24	Same as Ex
Direct exercise supervision	-	Weekly, weeks 1 to 12; Monthly, weeks 13 to 24	Same as Ex
Home exercise	-	Weeks 1 to 24	Same as Ex
Individual nutrition counseling	-	-	Weekly, weeks 1 to 12
ONS (approximately 270 kcal; 11 g protein; 0.6 g HMB; 0.3 g omega-3; 2.0 g omega-6; 5.25 g omega-9 fatty acids; and 300 IU vitamin D)	-	-	1 serving/day, weeks 1 to 12

Ex group, exercise intervention group; ONS, oral nutritional supplement; HMB, beta-Hydroxy-beta-methylbutyrate.

**Table 2:** Baseline characteristics of participants by study arm.

Characteristic	Control (*n* = 30)	Ex (*n* = 30)	Combined (*n* = 30)	*p*
Age (years), mean ± SD	65.7 ± 3.3	65.0 ± 3.1	65.7 ± 3.4	0.663
Sex, n (%)				0.018
Male	14 (46.7)	5 (16.7)	6 (20.0)	
Female	16 (53.3)	25 (83.3)	24 (80.0)	
Educational level, n (%)				0.722 *
Primary school	20 (66.7)	15 (50.0)	21 (70.0)	
Secondary school	5 (16.7)	8 (26.7)	4 (13.3)	
High school	4 (13.3)	4 (13.3)	3 (10.0)	
Higher education ^a^	1 (3.3)	3 (10.0)	2 (6.7)	
Employment status, n (%)				0.303
Not employed	25 (83.3)	20 (66.7)	21 (70.0)	
Currently working	5 (16.7)	10 (33.3)	9 (30.0)	
Cohabitation, n (%)				>0.99 *
Yes	27 (90.0)	26 (86.7)	26 (86.7)	
No	3 (10.0)	4 (13.3)	4 (13.3)	
Low family economics ^b^, n (%)				0.421
Yes	10 (33.3)	6 (20.0)	10 (33.3)	
No	20 (66.7)	24 (80.0)	20 (66.7)	
Smoking, n (%)				0.217 *
Yes	7 (23.3)	2 (6.7)	4 (13.3)	
No	23 (76.7)	28 (93.3)	26 (86.7)	
Multimorbidity ^c^, n (%)				0.824
Yes	12 (40.0)	10 (33.3)	10 (33.3)	
No	18 (60.0)	20 (66.7)	20 (66.7)	
Polypharmacy ^d^, n (%)				0.326 *
Yes, n (%)	0 (0.0)	2 (6.7)	0 (0.0)	
No, n (%)	30 (100.0)	28 (93.3)	30 (100.0)	
History of falls, n (%)				>0.99 *
Yes	4 (13.3)	3 (10.0)	4 (13.3)	
No	26 (86.7)	27 (90.0)	26 (86.7)	
Energy intake (kcal/day), mean ± SD	1479.6 ± 358.2	1338.5 ± 293.1	1410.7 ± 287.8	0.227
Protein intake (g/day), mean ± SD	62.7 ± 14.2	61.5 ± 14.6	61.9 ± 16.1	0.953

Ex group, exercise intervention group; SD, standard deviation; BMI, body mass index; SMI, skeletal muscle mass index. * Fisher’s exact test. ^a^ Participants were categorized as having higher educational levels if they graduated from university or graduate school. ^b^ Based on participants’ responses. ^c^ Multimorbidity was assessed using the Charlson Comorbidity Index, with a total score of ≥2 indicating the presence of multiple comorbid conditions [41,42,43]. ^d^ Participants were categorized as having polypharmacy if they concurrently used five or more medications (≥5 medications) [44].

**Table 3:** Baseline body composition, handgrip strength, and 6 m walking performance.

Variable	Control (*n* = 30)	Ex (*n* = 30)	Combined (*n* = 30)	*p*
Weight (kg)	51.8 ± 5.5	51.9 ± 6.6	52.9 ± 7.7	0.769
Body mass index (kg/m^2^)	21.6 ± 3.0	22.3 ± 3.1	22.7 ± 3.1	0.385
Fat mass (kg)	16.0 ± 6.6	17.5 ± 5.6	18.8 ± 6.3	0.205
Fat-free mass (kg)	35.9 ± 5.4	34.4 ± 4.5	34.1 ± 4.3	0.307
Skeletal muscle mass (kg)	19.0 ± 3.3	18.3 ± 2.7	17.9 ± 2.5	0.354
Skeletal muscle index (kg/m^2^)	5.7 ± 0.6	5.5 ± 0.5	5.4 ± 0.6	0.098
Handgrip strength (kg)	17.2 ± 7.9	17.2 ± 6.8	19.2 ± 5.3	0.404
Gait speed (m/s)	0.79 ± 0.19	0.95 ± 0.19	0.87 ± 0.18	**0.013**

Ex, exercise intervention. Results are presented as mean ± standard deviation. Bold *p*-values show significant differences between groups.

**Table 4:** Longitudinal changes in body composition, handgrip strength, and gait speed.

Variable	Group	T1 Mean ± SD	T2 Mean ± SD	Changes After 12 Weeks			Changes After 24 Weeks		
				Δ from T0 Estimate (95% CI)	p_w1_	p_b1_	Δ from T0 Estimate (95% CI)	p_w2_	p_b2_
Weight (kg)						0.338			0.291
	Control	52.0 ± 5.8	51.7 ± 6.1	0.1 (−0.3; 0.6)	0.552		−0.2 (−0.6; 0.3)	0.515	
	Ex	52.5 ± 6.6	52.2 ± 6.8	0.6 (0.1; 1.1)	0.010		0.4 (−0.1; 0.8)	0.118	
	Combined	53.5 ± 7.7	53.1 ± 8.1	0.5 (0.1; 1.0)	**0.030**		0.1 (−0.3; 0.6)	0.542	
BMI (kg/m^2^)						0.271			0.270
	Control	21.6 ± 3.1	21.5 ± 3.2	0.0 (−0.2; 0.2)	0.647		−0.1 (−0.3; 0.1)	0.557	
	Ex	22.6 ± 3.1	22.5 ± 3.2	0.3 (0.1; 0.5)	**0.009**		0.2 (−0.0; 0.4)	0.089	
	Combined	22.9 ± 3.1	22.7 ± 3.1	0.2 (0.0; 0.4)	**0.029**		0.1 (−0.1; 0.3)	0.514	
FM (kg)						0.677			0.366
	Control	16.2 ± 6.7	15.9 ± 6.9	0.3 (−0.2; 0.7)	0.277		−0.0 (−0.5; 0.4)	0.944	
	Ex	18.0 ± 5.8	17.8 ± 5.7	0.5 (0.1; 1.0)	**0.022**		0.3 (−0.1; 0.8)	0.146	
	Combined	19.2 ± 6.6	19.3 ± 6.6	0.3 (−0.1; 0.8)	0.167		0.4 (−0.0; 0.9)	0.069	
FFM (kg)						0.559			0.556
	Control	35.8 ± 5.6	35.7 ± 5.6	−0.1 (−0.5; 0.3)	0.580		−0.1 (−0.5; 0.3)	0.495	
	Ex	34.4 ± 4.4	34.4 ± 4.3	0.1 (−0.3; 0.5)	0.709		0.0 (−0.4; 0.4)	0.858	
	Combined	34.3 ± 4.2	33.8 ± 4.6	0.2 (−0.2; 0.6)	0.338		−0.3 (−0.7; 0.1)	0.177	
SMM (kg)						0.967			0.217
	Control	19.0 ± 3.3	19.1 ± 3.4	0.0 (−0.3; 0.2)	0.803		0.1 (−0.2; 0.3)	0.657	
	Ex	18.3 ± 2.7	18.3 ± 2.6	0.0 (−0.2; 0.2)	0.956		0.0 (−0.3; 0.2)	0.760	
	Combined	18.0 ± 2.5	17.7 ± 2.8	0.0 (−0.2; 0.2)	0.934		−0.2 (−0.5; 0.0)	**0.049**	
SMI (kg/m^2^)						0.173			**0.041**
	Control	5.7 ± 0.7	5.7 ± 0.7	−0.0 (−0.1; 0.0)	0.401		−0.1 (−0.1; −0.0)	**0.050**	
	Ex	5.5 ± 0.6	5.5 ± 0.5	0.1 (−0.0; 0.2)	0.093		0.0 (−0.0; 0.1)	0.191	
	Combined	5.5 ± 0.6	5.4 ± 0.7	0.0 (−0.0; 0.1)	0.262		−0.1 (−0.1; 0.0)	0.112	
HGS (kg)						**<0.001**			**<0.001**
	Control	16.3 ± 7.7	16.1 ± 7.5	−1.0 (−1.6; −0.3)	**0.004**		−1.1 (−1.8; −0.5)	**0.001**	
	Ex	19.5 ± 6.9	18.5 ± 6.5	2.4 (1.7; 3.0)	**<0.001**		1.3 (0.7; 2.0)	**<0.001**	
	Combined	22.1 ± 5.7	20.3 ± 5.3	2.9 (2.2; 3.6)	**<0.001**		1.1 (0.4; 1.7)	**<0.001**	
Gait speed ^†^ (m/s)						**<0.001**			**<0.001**
	Control	0.78 ± 0.19	0.79 ± 0.19	−0.02 (−0.07; 0.03)	0.511		−0.01 (−0.06; 0.04)	0.667	
	Ex	1.21 ± 0.28	1.15 ± 0.27	0.26 (0.21; 0.31)	**<0.001**		0.20 (0.15; 0.24)	**<0.001**	
	Combined	0.98 ± 0.17	0.91 ± 0.18	0.11 (0.05; 0.15)	**<0.001**		0.04 (−0.01; 0.09)	0.082	

Ex group, exercise intervention group; BMI, body mass index; FM, fat mass; FFM, fat-free mass; SMM, skeletal muscle mass; SMI, skeletal muscle index; HGS, handgrip strength; CI, confidence interval; SD, standard deviation. T0: baseline; T1: 12 weeks; T2: 24 weeks. Models were adjusted for sex. Δ: model-estimated mean change from baseline. p_w_: *p*-value for the within-group contrast between each follow-up time point and baseline, derived from the linear mixed-effects model. p_b_: *p*-value for the overall group-by-time interaction at each follow-up, testing differences in change from baseline among study groups. ^†^ Estimates are additionally adjusted for baseline gait speed; higher gait speed and positive change indicate better performance. Bold *p*-values show significant differences between groups.

**Table 5:** Adjusted pairwise between-group comparisons at 12 and 24 weeks.

Variable	Time	Ex vs. Control Adjusted Pairwise Estimate (95% CI)	*p*	Combined vs. Control Adjusted Pairwise Estimate (95% CI)	*p*	Combined vs. Ex Adjusted Pairwise Estimate (95% CI)	*p*
Weight (kg)	T1	0.47 (−0.19; 1.14)	0.164	0.38 (−0.29; 1.05)	0.264	−0.09 (−0.76; 0.57)	0.784
	T2	0.53 (−0.13; 1.20)	0.117	0.30 (−0.36; 0.97)	0.373	−0.23 (−0.90; 0.44)	0.499
BMI (kg/m^2^)	T1	0.22 (−0.06; 0.50)	0.127	0.18 (−0.11; 0.46)	0.221	−0.04 (−0.33; 0.24)	0.764
	T2	0.23 (−0.05; 0.52)	0.106	0.13 (−0.16; 0.41)	0.380	−0.11 (−0.39; 0.18)	0.460
FM (kg)	T1	0.28 (−0.37; 0.94)	0.397	0.07 (−0.59; 0.73)	0.834	−0.21 (−0.87; 0.44)	0.523
	T2	0.36 (−0.30; 1.02)	0.281	0.45 (−0.21; 1.10)	0.181	0.09 (−0.57; 0.74)	0.795
FFM (kg)	T1	0.19 (−0.38; 0.76)	0.512	0.31 (−0.26; 0.88)	0.285	0.12 (−0.45; 0.69)	0.679
	T2	0.18 (−0.39; 0.75)	0.542	−0.14 (−0.71; 0.43)	0.637	−0.31 (−0.88; 0.26)	0.280
SMM (kg)	T1	0.04 (−0.30; 0.37)	0.829	0.04 (−0.29; 0.37)	0.814	0.00 (−0.33; 0.34)	0.984
	T2	−0.09 (−0.42; 0.24)	0.596	−0.29 (−0.62; 0.04)	0.088	−0.20 (−0.53; 0.13)	0.239
SMI (kg/m^2^)	T1	0.09 (−0.01; 0.19)	0.075	0.07 (−0.03; 0.17)	0.166	−0.02 (−0.12; 0.08)	0.692
	T2	0.12 (0.02; 0.22)	**0.021**	0.01 (−0.09; 0.11)	0.792	−0.10 (−0.20; −0.00)	**0.041**
HGS (kg)	T1	3.33 (2.41; 4.26)	**<0.001**	3.85 (2.93; 4.77)	**<0.001**	0.52 (−0.41; 1.44)	0.273
	T2	2.49 (1.57; 3.42)	**<0.001**	2.23 (1.30; 3.15)	**<0.001**	−0.27 (−1.19; 0.66)	0.572
Gait speed ^†^ (m/s)	T1	0.28 (0.21; 0.35)	**<0.001**	0.12 (0.05; 0.19)	**<0.001**	−0.16 (−0.22; −0.09)	**<0.001**
	T2	0.21 (0.13; 0.28)	**<0.001**	0.05 (−0.02; 0.12)	0.137	−0.15 (−0.22; −0.08)	**<0.001**

Ex, exercise intervention; BMI, body mass index; FM, fat mass; FFM, fat-free mass; SMM, skeletal muscle mass; SMI, skeletal muscle index; HGS, handgrip strength; CI, confidence interval; T1, 12 weeks; T2, 24 weeks. Estimates are sex-adjusted pairwise differences in change from baseline. ^†^ Estimates are additionally adjusted for baseline gait speed; higher values indicate better performance. Bold *p*-values show significant differences between groups.

## Data Availability

The original contributions presented in this study are included in the article. Further inquiries can be directed to the corresponding author. The study was retrospectively registered, with the clinical trial number NCT07761091, on 12 August 2026.

## Abbreviations

The following abbreviations are used in this manuscript:

AWGS	Asian Working Group for Sarcopenia
BIA	Bioelectrical impedance analysis
BMI	Body mass index
CI	Confidence interval
Ex	Exercise intervention
FFM	Fat-free mass
FM	Fat mass
HGS	Handgrip strength
HMB	beta-Hydroxy-beta-methylbutyrate
MM	Muscle mass
ONS	Oral nutritional supplement
SD	Standard deviation
SMI	Skeletal muscle index
SMM	Skeletal muscle mass
